# Clinical Evaluation, Patch Testing, and Quality of Life Assessment in Patients With Cosmetic Dermatitis

**DOI:** 10.7759/cureus.75503

**Published:** 2024-12-10

**Authors:** Harshavardhan Jampuram, Aneet Mahendra, Sanjeev Gupta

**Affiliations:** 1 Dermatology, Maharishi Markandeshwar Institute of Medical Sciences and Research, Ambala, IND

**Keywords:** contact dermatitis, cosmetics, dlqi, patch test, quality of life

## Abstract

Background

Cosmetics have become an integral part of the contemporary lifestyle. Contact dermatitis (CD) is an inflammatory skin disease resulting from exposure to an external chemical present in cosmetics. A patch test is considered the criterion standard method for detecting CD. CD has been demonstrated to be detrimental to the quality of life (QoL).

Objective

The present study aims to investigate the clinical-epidemiological profile of CD and identify the possible allergens involved in it through patch testing. We also aim to assess the impact of QoL on patients presenting with CD.

Methodology

A total of 65 patients with CD were enrolled in the study. A comprehensive history of cosmetic products was obtained, along with dermatological and systemic examination. Patch testing was done to identify possible allergens with the Indian Cosmetic and Fragrance series and allergens from the Indian Standard Battery as and when required. A quality-of-life assessment was done using the Dermatology Life Quality Index (DLQI) questionnaire.

Results

The mean age of the patients was 43.2 ± 11.9 years, with a high preponderance of females (40; 61.5%). The most common cosmetic used was hair dye (45; 69.2%), followed by moisturizer/lotion/cream (23; 35.4%) and soap/shampoo/cleanser (16; 24.6%). The most prevalent site of allergy was the face (53; 81.5%), followed by the scalp (35; 53.8%), eyelids (33; 50.8%), and neck (22; 33.8%). The most common allergen was para-phenylenediamine (22; 33.8%), followed by fragrance mix (10; 15.4%) and Kathon CG (methylchloroisothiazolinone + methylisothiazolinone) (8; 12.3%). The mean DLQI score was 8.61 ± 5.36. Most patients had mild to moderate DLQI scores (45; 69.2%).

Conclusion

The present study was a prospective study that analyzed the clinical-epidemiological profile and impairment of QoL in CD. We identified the possible allergens involved in cosmetic CD by patch testing with the Indian cosmetic and fragrance series. We also assessed the impact on the quality of life in patients with cosmetic dermatitis. Further multicentric studies with ample sample sizes are required to validate the findings of this study.

## Introduction

The Federal Food, Drug, and Cosmetic Act (FD&C Act) defines cosmetics as articles or items that are meant to be applied to the body of humans for cleansing, beautifying, and enhancing appearance and attractiveness. These items can be poured, rubbed, sprayed, sprinkled, or otherwise applied to the body. This term encompasses a wide range of items, such as moisturizers for the skin, lipsticks, perfumes, nail polishes, eye and face cosmetics, shampoos, hair color, permanent waves, toothpaste, deodorants, and any material intended to be used as an ingredient in a cosmetic product [1]. Cosmetics have become an integral part of the modern lifestyle. Their use has physical and psychological effects, which help people improve their looks and self-esteem. With the over-the-counter availability of numerous products, their use goes unchecked, and without the knowledge of the constituents and their mechanism of action, prolonged use can often lead to adverse effects [1].

Contact dermatitis (CD) is an inflammatory skin disease that follows exposure to an external chemical. There are two subtypes of CD, irritant CD (ICD) and allergic CD (ACD), which vary in both clinical and pathophysiological aspects [2]. Other types of CDs, such as photo contact dermatitis, include phototoxic and photoallergic CDs. Additionally, mucous membranes may be affected. The most common kind of CD, known as ICD (around 80% of cases of CD), is brought on by inflammatory and cytotoxic results from exposure to various environmental factors (chemical or physical) that might trigger the innate immune system [3]. In ACD, the innate immune system is activated once an allergen penetrates the skin and is captured by the Langerhans cells (LC), leading to antigen recognition and proliferation of antigen-specific CD4+ and CD8+ T-cells in the lymph node. Further, keratinocyte damage and death occur on residual or reexposure to the allergen mediated by the antigen-specific T-cells. 

A patch test is considered the gold standard method for detecting CD. Using a systematic approach to imitate the triggering phase of type IV hypersensitivity, a specific allergen is given to the skin under occlusion. On day 2 (D2), 48 hours after applying the patch test chambers on D0, removing the chambers as per the Italian Guidelines for Patch Testing is necessary. At D2, reading is obtained between fifteen to sixty minutes after removing the chambers. At either D3 or D4, a second reading is required. Some specific allergies, such as corticosteroids and aminoglycoside medications, need a third assessment to be conducted between the fifth and tenth day [4].

Quality of life (QoL) measures are essential to assessing the benefits of dermatology therapies, particularly for long-term, incurable conditions. The body of research has demonstrated that CD has a detrimental effect on QoL. Several characteristics, such as itching, pain, and difficulty using hands or doing daily tasks, are linked to worsening QoL. When dermatological conditions have a long-term adverse effect on QoL, it can lead to significant emotional and functional disability. The degree of CD impact on QoL is not necessarily commensurate with the severity of the illness, maybe because of the psychological strain and remorse associated with the disease's visual symptoms [5].

The impact and effect of different skin conditions are measured using a standard questionnaire called the Dermatology Life Quality Index (DLQI). DLQI scores can be impacted by several variables, including age group, sex, frequency of exacerbations, and severity of the condition [6]. The present study is undertaken to observe clinical manifestations and patch testing to identify the possible allergens in cosmetics causing adverse effects and impact on QoL.

## Materials and methods

Study design

The present study was an observational and cross-sectional study that included 65 patients with cosmetic contact dermatitis who attended the Dermatology Outpatient Department at the Maharishi Markandeshwar Institute of Medical Sciences and Research, Mullana, Ambala, Haryana, over a period of one year and six months from January 2023 to June 2024.

Inclusion criteria

Patients of either sex above 12 years of age (if <18 years with consent of the parent or guardian) with a clinically suspected case of contact dermatitis due to cosmetics, patients willing to undergo patch testing, and patients with dermatitis in the quiescent stage.

Exclusion criteria

Patients diagnosed with ICD, patients clinically showing signs of immunosuppression and severe concomitant skin disorders, patients who are taking steroids (prednisolone, at doses more than 15-20 mg/day) due to dermatological or any other problem, pregnant and lactating females, and patients in whom patch test strips cannot be applied because of a local problem at the site.

Participants were thoroughly explained the study objectives, and proper consent was obtained. With due permission from the Institutional Ethical Committee of Maharishi Markandeshwar Institute of Medical Sciences and Research (IEC: 2548), 65 clinically suspected patients of allergic contact dermatitis to cosmetics were included.

Data collection

A proper, well-explained consent form was obtained from all the study subjects regarding the patch test in the regional language. A thorough history of sociodemographic determinants, cosmetic used, and site of allergy was taken along with dermatological and systemic examinations. Patch testing was done to identify the possible allergens with the Indian Cosmetic and Fragrance series (by Systopic Laboratories Private Limited, New Delhi, India) and allergens from the Indian Standard Battery as and when required.

Quality of life assessment

QoL was measured using DLQI. The DLQI is a self-administered validated questionnaire to assess the quality of life of dermatology patients with ten questions covering the last seven days (Appendix 1). They measure the dimensions of health, such as symptoms and perceptions, daily activities, recreation, work/study, interpersonal relationships, sexuality, and treatment. Each question has a Likert-type scale with four answer options: 0: not at all, 1: a little, 2: a lot, 3: very much. The final score is obtained by summing the score of each item and ranges from 0 (no impact on QoL) to 30 points (maximum impact on QoL). Interpretation of DLQI score 0-1 (no effect at all on patient's life), 2-5 (mild/small effect), 6-10 (moderate effect), 11-20 (high/very large effect), and 21-30 (very high/extremely large effect).

Statistical analysis

The data collected was entered into an Excel worksheet. Data was analyzed using the IBM SPSS Statistics for Windows, Version 27 (Released 2020; IBM Corp., Armonk, New York, United States). The data is presented in the form of a number and a fraction of the total. Total frequency is divided into various subgroups for certain variables, and the number and percentage of cases belonging to each group are calculated. Appropriate tables and graphs are used to represent the data.

## Results

The mean age of the patients was 43.2 ± 11.9 years among the total study population. The most commonly affected age group was 41-50 years, with 19 (29.2%) patients, followed by the age group of 31-40 years, with 15 (23.1%) patients. The least affected age group was 61-70 years, with five (7.7%) patients. There were 40 (61.5%) females and 25 (38.5%) males in this study, indicating a female preponderance with a male-to-female ratio of 1:2.6. Out of 65 patients, 37 (56.9%) patients belong to urban areas, whereas 28 (43.1%) patients come from rural areas, indicating that the majority of patients were residing in urban areas. In a total of 65 patients, 58 (89.2%) patients were married, whereas the rest of the seven (10.78%) patients were unmarried. Most of the patients were homemakers (33; 50.8%), followed by private jobs (salaried employees) (14; 21.5%) and students (9; 13.8%). Out of 65 patients, 22 (33.8%) patients had obtained a high school level education, followed by 18 (27.7%) patients who had obtained a middle school level education, and 16 (24.6%) patients were graduates. The least common was primary school level education obtained by one (1.5%) patient (Table 1). 

**Table 1:** Sociodemographic determinants of the enrolled patients.

Variable	Domain	Number	Percentage (%)
Age distribution	Mean age	43.15 ± 11.88 Yrs	
	21-30 Yrs	13	20.0
	31-40 Yrs	15	23.1
	41-50 Yrs	19	29.2
	51-60 Yrs	13	20.0
	61-70 Yrs	5	7.7
Gender distribution	Female	40	61.5
	Male	25	38.5
Residence	Urban	37	56.9
	Rural	28	43.1
Marital status	Married	58	89.2
	Unmarried	7	10.8
Occupation	Homemakers	33	50.8
	Private jobs	14	21.5
	Students	9	13.8
	Farmers	4	6.2
	Laborers	3	4.6
	Self-employed	2	3.1
Education	Illiterate	8	12.3
	Primary school	1	1.5
	Middle school	18	27.7
	High school	22	33.8
	Graduate	16	24.6

**Figure 1:**
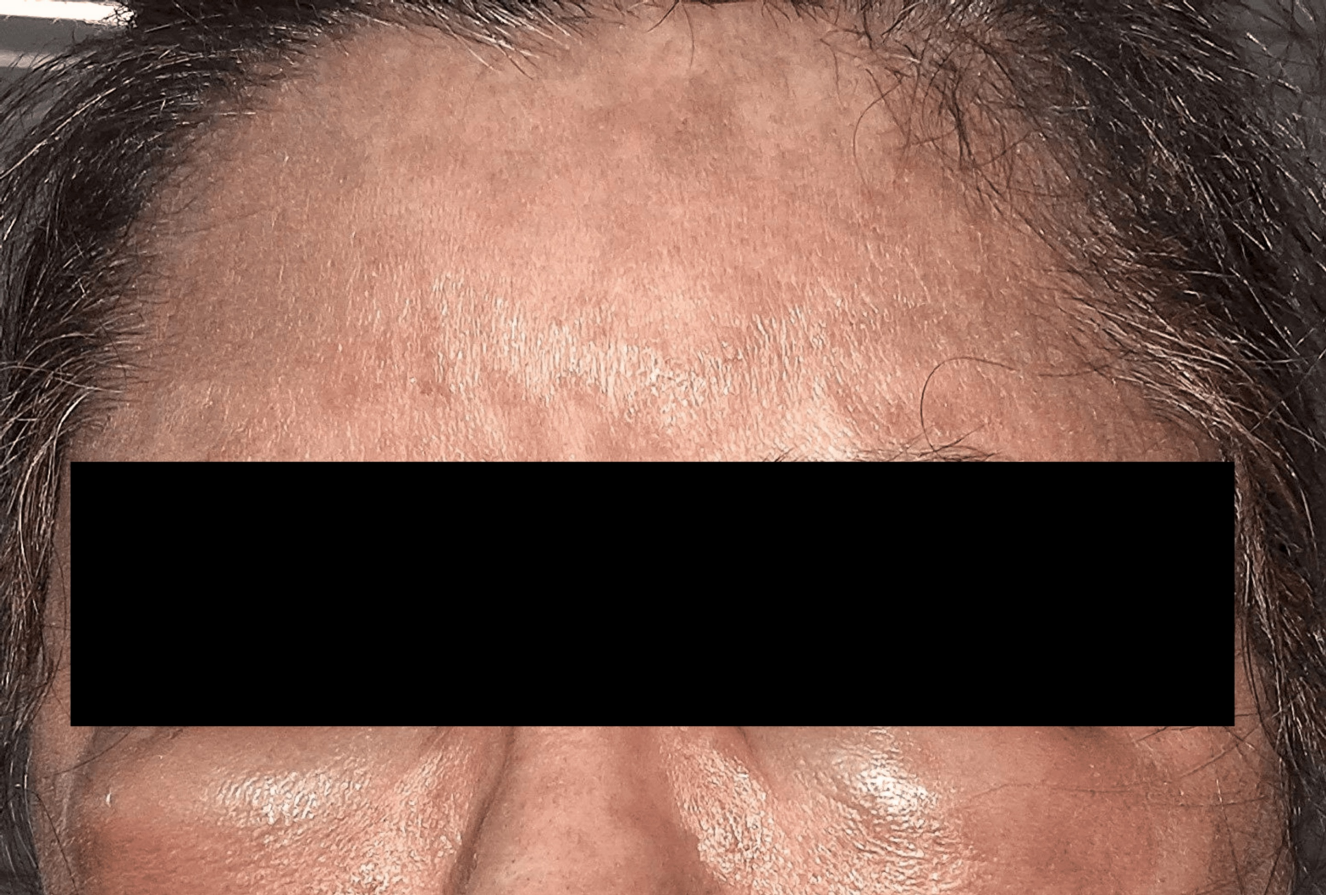
Diffuse erythema and facial edema developing after hair dye application.

**Figure 2:**
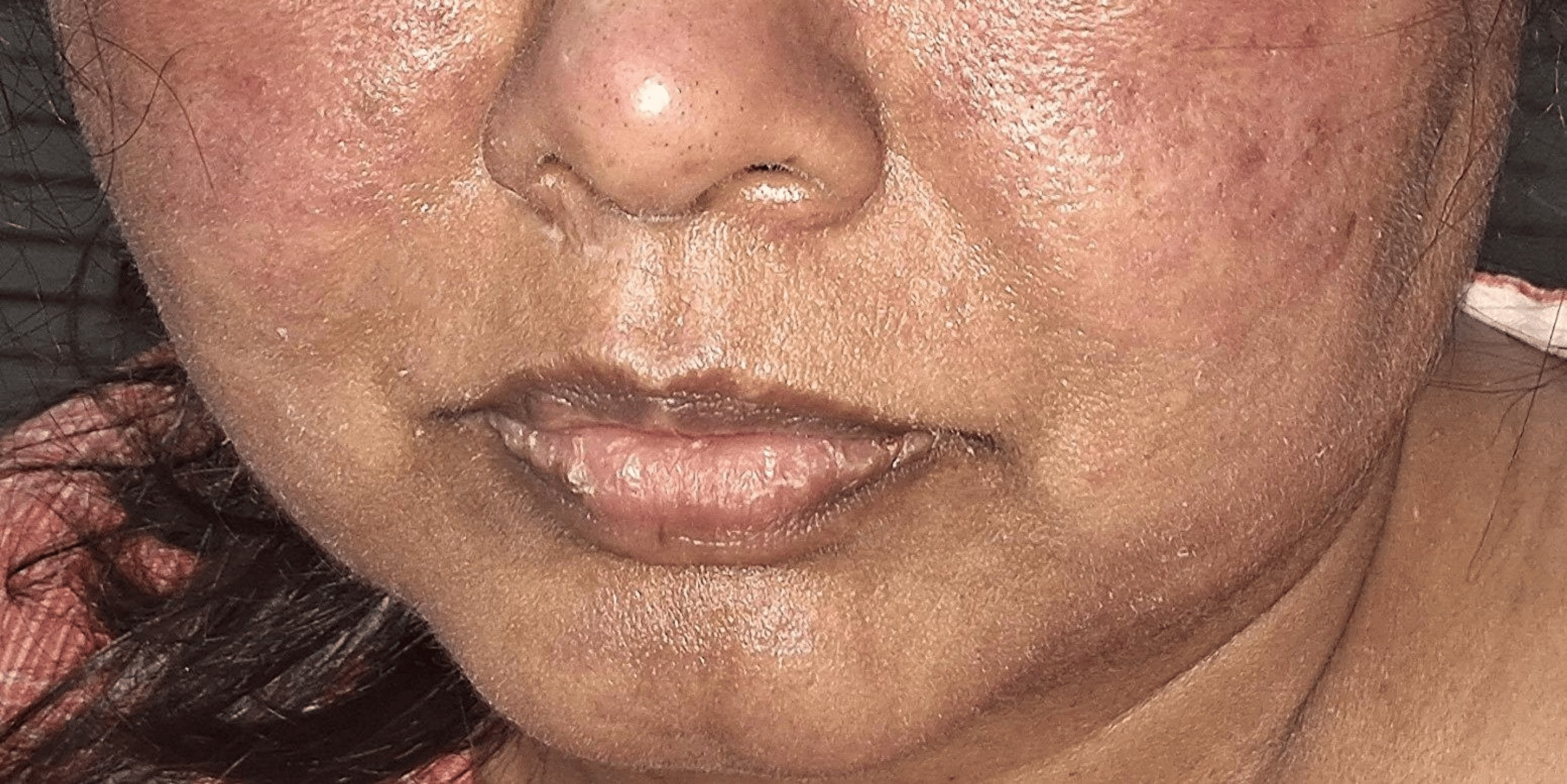
Diffuse erythema and facial edema developing after hair dye application.

**Figure 3:**
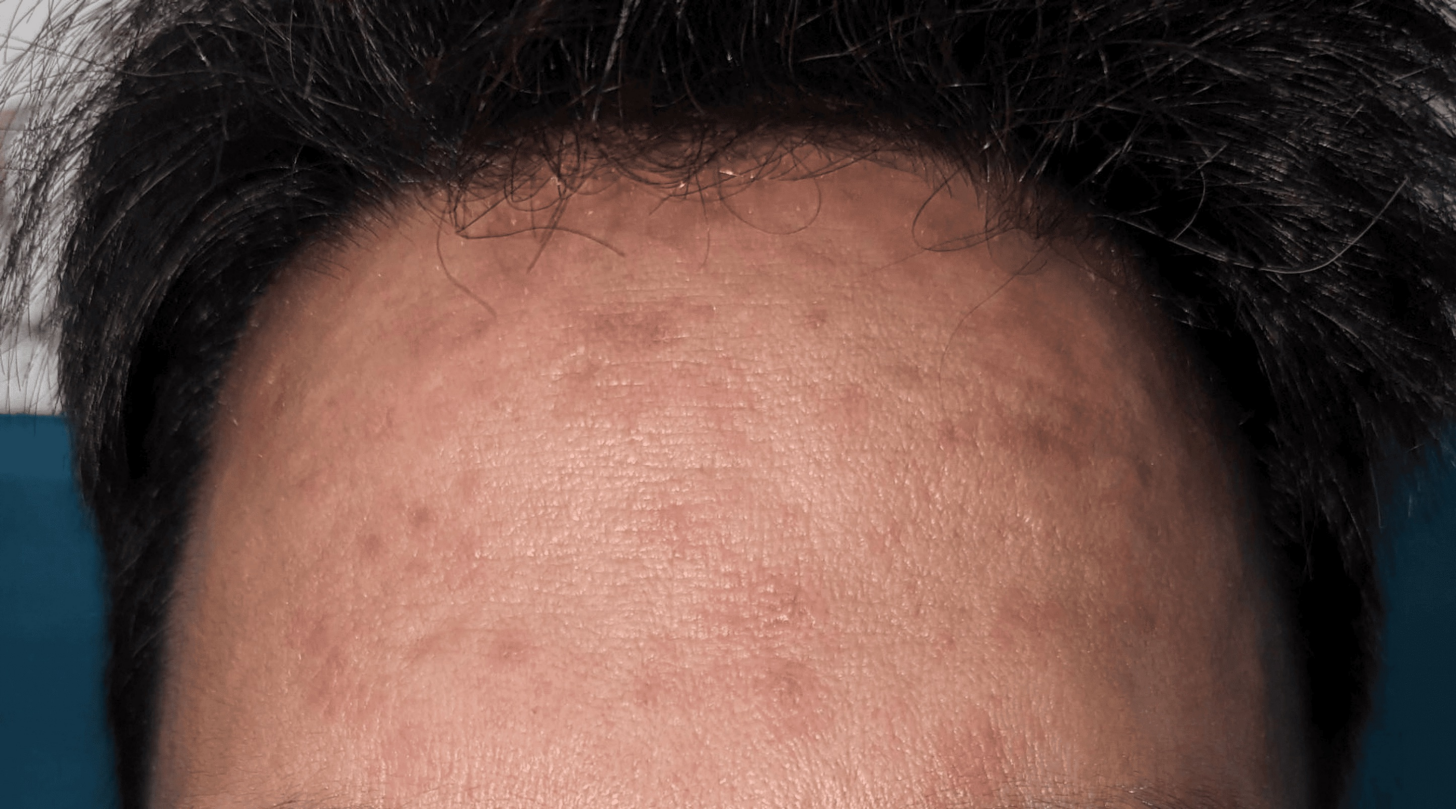
Maculopapular lesions over the forehead with a history of face cream application.

**Figure 4:**
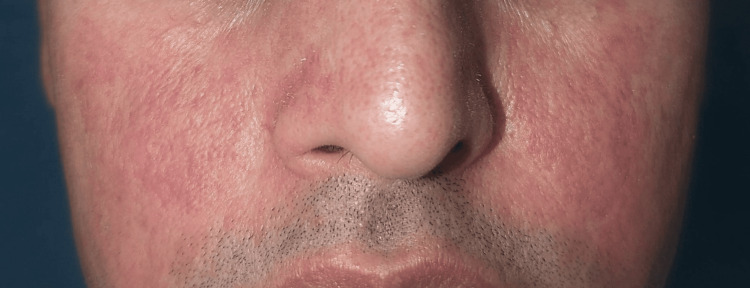
Diffuse erythema over bilateral cheeks with a history of face cream application.

**Figure 5:**
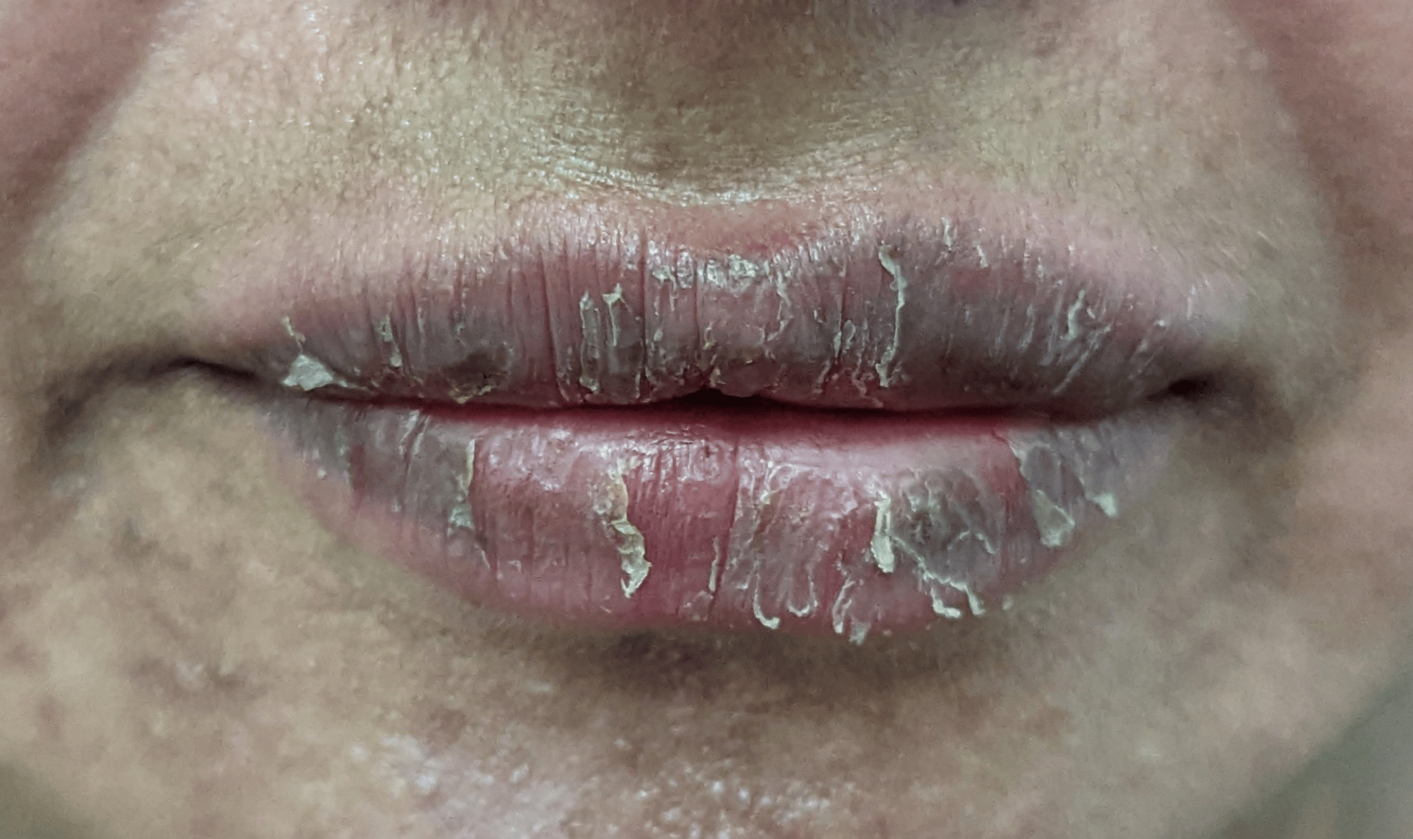
Erythema with scaling over lips with a history of lipstick application.

**Figure 6:**
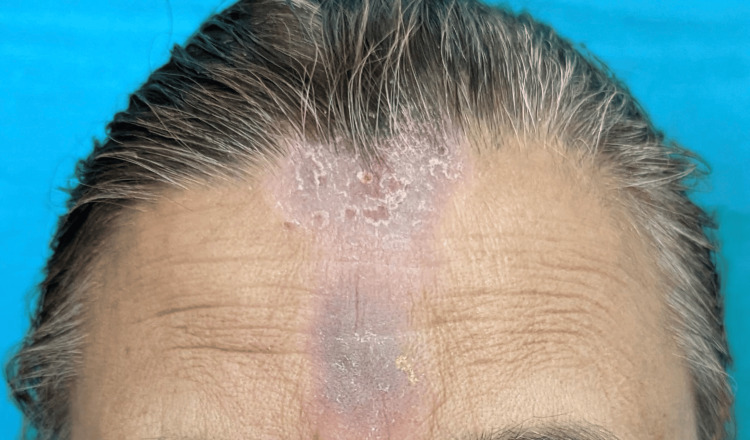
Hyperpigmentation and scaling with surrounding erythema at the site of sindoor application.

**Figure 7:**
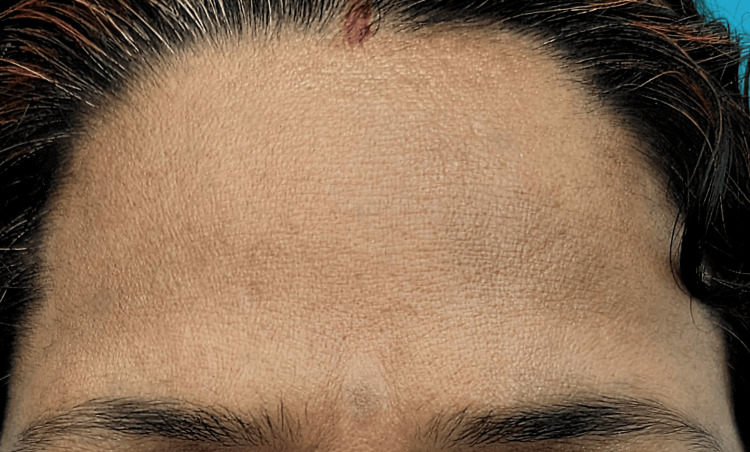
Hyperpigmentation with peripheral hypopigmentation at the site of glued bindi application.

**Figure 8:**
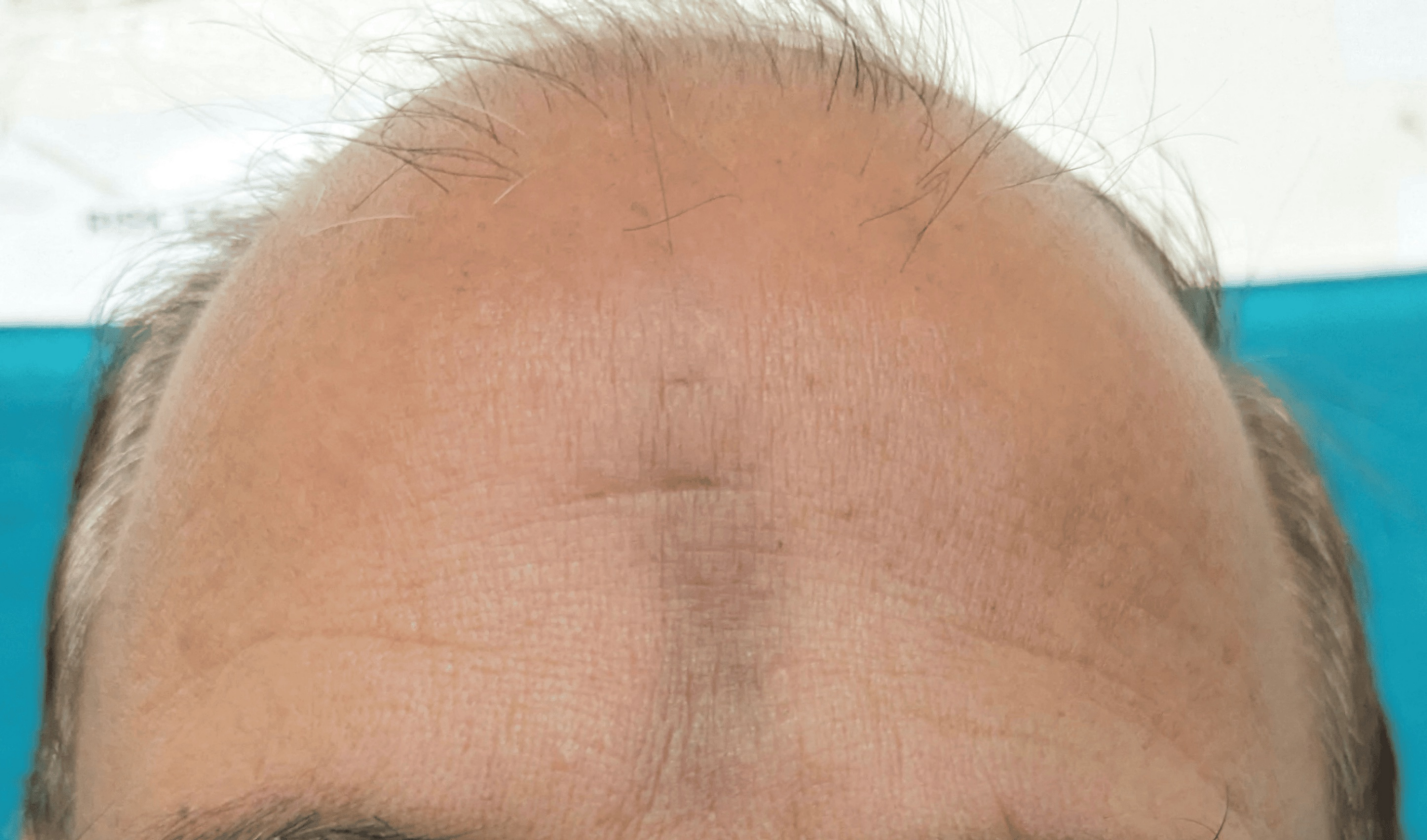
Hyperpigmentation at the site of Chandan/sandalwood application.

The most commonly used cosmetic product was hair dye (45; 69.2%) (Figure 1, Figure 2), followed by moisturizer/lotion/cream (23; 35.4%) (Figure 3, Figure 4), soap/shampoo/cleanser (16; 24.6%), lipstick/lip balm (9; 13.8%) (Figure 5), perfume/fragrance (6; 9.2%), sindoor/kumkum (6; 9.2%) (Figure 6), bindi (5; 7.7%) (Figure 7), nail cosmetics (1; 1.5%), and Chandan/sandalwood (1; 1.5%) (Figure 8) (Table 2). 

**Table 2:** List of different cosmetics used by patients.

Cosmetic used	Number	Percentage
Hair dye	45	69.2
Moisturizer/lotion/cream	23	35.4
Soap/shampoo/cleanser	16	24.6
Lipstick/lip balm	9	13.8
Perfume/fragrance	6	9.2
Sindoor/kumkum	6	9.2
Bindi	5	7.7
Nail cosmetics	1	1.5
Chandan/sandalwood	1	1.5

The most common site of allergy was the face (53; 81.5%), followed by the scalp (35; 53.8%), eyelids (33; 50.8%), neck (22; 33.8%), lips (11; 16.9%), trunk (8; 12.3%), and upper limbs (7; 10.8%). The patch test was positive in 43 (66.1%) patients. One allergen was positive in 24 (36.9%) patients, two allergens were positive in 11 (16.9%) patients, three allergens were positive in six (9.2%) patients, and more than three allergens were positive in two (3.1%) patients.

The most common positive allergen was para-phenylenediamine (22; 33.8%), followed by fragrance mix (10; 15.4%), Kathon CG (8; 12.3%), sindoor/kumkum (5; 7.7%), cetrimide (4; 6.2%), Peru balsam/balsam of Peru (3; 4.6%), wool alcohol (lanolin) (3; 4.6%), nickel sulphate (2; 3.1%), thiuram mix (2; 3.1%), bindi (2; 3.1%), parabens mix (2; 3.1%), potassium dichromate (2; 3.1%), formaldehyde (2; 3.1%), colophony (2; 3.1%), geranium oil (2; 3.1%), copper sulphate (2; 3.1%), butyl hydroxytoluene (BHT) (1; 1.5%), parthenium (1; 1.5%), lavender absolute (1; 1.5%), epoxy resins (1; 1.5%), and musk mix (1; 1.5%) (Table 3).

**Table 3:** Different allergens positivity on patch tests.

Positive allergens	Number	Percentage
Para-phenylenediamine	22	33.8
Fragrance Mix	10	15.4
Kathon CG	8	12.3
Sindoor/kumkum	5	7.7
Cetrimide	4	6.2
Peru balsam/balsam of Peru	3	4.6
Wool alcohol (Lanolin)	3	4.6
Nickel sulphate	2	3.1
Thiuram mix	2	3.1
Bindi	2	3.1
Parabens mix	2	3.1
Potassium dichromate	2	3.1
Formaldehyde	2	3.1
Colophony	2	3.1
Geranium oil	2	3.1
Copper sulphate	2	3.1
Butyl hydroxytoluene (BHT)	1	1.5
Parthenium	1	1.5
Lavender absolute	1	1.5
Epoxy resins	1	1.5
Musk mix	1	1.5

The mean DLQI score was 8.61 ± 5.36. The DLQI score indicated that the quality of life was impaired to a mild level in 24 (36.9%) patients, to a moderate level in 21 (32.3%) patients, to a high level in 18 (27.7%) patients, and to a very high level in two (3.1%) patients. Most patients had mild to moderate DLQI scores (45; 69.2% of patients).

**Figure 9:**
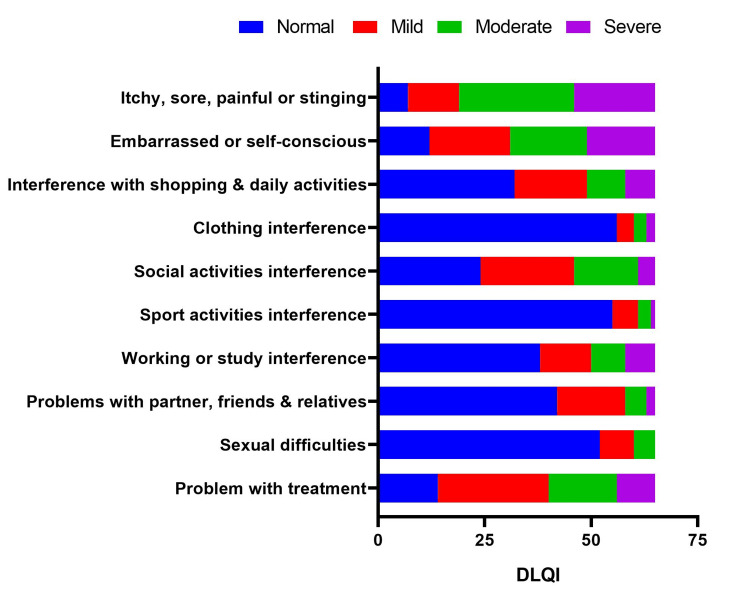
Responses to the DLQI questionnaire. DLQI: Dermatology Life Quality Index

Around 29.2% of patients reported severe itching, soreness, pain, or stinging; 24.6% reported embarrassment or self-consciousness; and 10.8% reported shopping interference (Figure 9).

## Discussion

Inflammation of the skin brought on by chemicals coming into contact with the skin is known as contact dermatitis (CD). CD has a genetic predisposition, and antecedent exposure is necessary for the type IV delayed hypersensitivity reaction [7]. The clinical symptoms of CD are influenced by the intensity and frequency of exposure to the allergen, the specific type of allergen, and several factors connected to the individual affected. Each patient has a different clinical presentation, frequently presenting a diagnostic difficulty to the treating dermatologist [8]. Patch testing is a highly useful outpatient department (OPD)-based method for identifying the allergens in consideration [7]. This study on patch testing with cosmetic allergens helps detect contact dermatitis early on and treat the condition before it worsens, saving money and significantly enhancing patient quality of life.

The mean age of the patients in the present study was 43.15 ± 11.88 years. Similarly, in the previous studies, the mean age of the study population was Boonchai et al., 43.2 ± 16.7 years [6], Yadav et al., 42.28 ± 13 years [7]. People in the fourth and fifth decade of life may have a high prevalence of contact dermatitis due to their increased use of cosmetic products to maintain a youthful appearance and conceal signs of aging. This study comprised 40 females (61.5%) and 25 males (38.5%), indicating a predominance of females with a male-to-female ratio of 1:2.6. Similarly, in the previous study by Boonchai et al., there were 379 (73.0%) female and 140 (27.0%) male patients [6]. In a recent study by Yadav et al., there were 39 female and 19 male patients [7]. Current and past studies likely attribute the high female predominance to women's increased exposure to cosmetic products. Women use face cleansers, deodorants, fragrances, and hair dyes more frequently than men.

Multiple studies assessing risk factors for facial and allergic contact dermatitis in general indicated that occupational exposure was the predominant cause [7]. In our study, most of the patients were homemakers (33; 50.8%), followed by private jobs (14; 21.5%) and students (9; 13.8%). In a recent study by Yadav et al., the majority of patients, accounting for 46.55%, were housewives, followed by businesspeople (10.3%) and farmers (8.6%) [7]. In the study by Hassan et al., the research population consisted of individuals from various occupations, including farmers, housewives, construction workers, and students [8]. Homemakers comprise nearly half of all cosmetic dermatitis cases in these studies. We could attribute this to their heightened use of cosmetics and regular exposure to domestic cleaning products. We observed no correlation between the onset of cosmetic contact dermatitis and work. However, it may occur less commonly in those with lower socioeconomic status, perhaps due to lower cosmetic use.

The most prevalent cosmetics used were hair dye (45; 69.2%), followed by moisturizer/lotion/cream (23; 35.4%), soap/shampoo/cleanser (16; 24.6%), lipstick/lip balm (9; 13.8%), and perfume/fragrance (6; 9.2%). In a recent study by Yadav et al., the most prevalent cosmetics used were soap (with 73.68% males and 64.10% females using it), face creams (with 42.10% males and 69.23% females using it), and hair color (with 63.15% males and 53.84% females using it) [7]. In a study by Mehta et al., the primary culprits responsible for face dermatitis, which occurs most frequently, have been associated with bindi, hair color, facial moisturizers, and creams [9]. Goel et al. found that scents and perfumes were the primary culprits of face CD in 40% of the individuals in their research, with soaps and shampoos being the second most prevalent cause at 28% [10].

The face was the most prevalent location for allergies, affecting 53 (81.5%) individuals, followed by the scalp (35; 53.8%), eyelids (33; 50.8%), and neck (22; 33.8%). In a previous study by Boonchai et al., the predominant sites of lesions were the face (39.7%) and hands (29.5%) [6]. In a recent study by Yadav et al., 65.78% of the patients had both facial and extra-facial involvement, whereas 34.21% exclusively showed facial involvement [7]. In the study by Hassan et al., the hands were the primary location in 268 instances (46.05%), followed by the feet, occurring in 81 cases (13.92%), and both the hands and feet (12.03%) [8]. In the study by Korkmaz and Boyvat, the most prevalent sites of allergy were the hands (31.4%), followed by the face, feet, and other sites [11]. In this study, the face is the most commonly affected site due to its frequent application of cosmetics.

The patch test yielded positive results in 43 (66.1%) individuals in the current study. Yadav et al. conducted recent research where 38 out of the 58 individuals studied (65.52%) showed positive patch-test findings. In Hassan et al.'s study, a total of 177 people with allergies tested positive (30.4%) for different allergens [8]. The increased prevalence of positive patch-test results in our study, as well as in other research, may be attributed to the fact that these investigations were done in bigger metropolitan areas where the usage of cosmetics is far more prevalent, particularly among women.

Our study found that 11 patients (16.9%) tested positive for two allergens, six patients (9.2%) tested positive for three allergens, and two patients (3.1%) tested positive for more than three allergens. A recent study by Yadav et al. showed that 14 out of 38 patients (36.8%) exhibited multiple antigen positivity. Among these patients, 11 showed positivity to two allergens, two showed positivity to three allergens, and one showed positivity to six allergens [7]. The study conducted by Hassan et al. showed that 38 patients had a positive reaction to two specific allergens, while the remaining 19 patients showed more than two positive patch test reactions [8].

In our study, the most common allergens were para-phenylenediamine (22; 33.8%), followed by fragrance mix (10; 15.4%), Kathon CG (8; 12.3%), sindoor/kumkum (5; 7.7%), cetrimide (4; 6.2%), Peru balsam/balsam of Peru (3; 4.6%), wool alcohol (lanolin) (3; 4.6%), nickel sulfate (2; 3.1%), thiuram mix (2; 3.1%), bindi (2; 3.1%), parabens mix (2; 3.1%), potassium dichromate (2; 3.1%), formaldehyde (2; 3.1%), colophony (2; 3.1%), geranium oil (2; 3.1%), copper sulfate (2; 3.1%), butyl hydroxytoluene (BHT) (1; 1.5%), parthenium (1; 1.5%), lavender absolute (1; 1.5%), epoxy resins (1; 1.5%), and musk mix (1; 1.5%). The study conducted by Yadav et al. revealed that thiomersal was the most prevalent allergen, accounting for 17.3% of cases, followed by fragrance mix (15.6%), para-phenylenediamine (12.1%), parthenium (10.3%), cetrimide (6.9%), colophony (5.2%), nickel (5.2%), geranium oil (3.5%), musk mix (3.5%), thiuram mix (3.5%), and potassium dichromate (3.5%) [6]. The study conducted by Hassan et al. revealed that nickel sulfate was the most prevalent allergen found in 49 cases, followed by potassium dichromate in 28 cases, cobalt sulfate in 26 cases, PPD in 23 cases, neomycin sulfate in 16 cases, and scent mix in 15 cases [8].

PPD was the allergen that most frequently caused positive reactions (22; 33.8%) in the current study. Towards the end of the 1800s, it was developed for use in hair color. Because of its longer-lasting nature and ability to give hair a black, natural-looking coloring, it is a desirable ingredient in many hair colors. To get the black hue, PPD needs a secondary component, such as an oxidizer or developer. PPD is oxidized to an allergic hapten in the epidermis or dermis. For many years, PPD has been recognized as a prevalent allergen associated with hair dye exposure [12]. The increasing frequency of hair color usage correlates with an increase in sensitivity to PPD.

Fragrance mix is the second (10; 15.4%) most recognized allergy. Eight components comprise the typical patch testing set, which may accurately identify up to 70% of all perfume allergies. The preservatives found in perfumes associated with ACD include formaldehyde, formaldehyde releasers, and non-formaldehyde releasers. These may be found in practically every cosmetic preparation [13].

Kathon CG was the third (8; 12.3%) most identified allergen. It is a mixture of methylchloroisothiazolinone and methylisothiazolinone and is often used as a preservative in home and industrial cleaning products, paints, adhesives, and cosmetics. The increased frequency of Kathon CG positives in women may be linked to sensitization brought on by regular cosmetic usage and cleaning products used in the household [14].

The present study's average DLQI score was 8.61 ± 5.36, indicating a mild to moderate degree (69.2%) of impact. This score is similar to the DLQI values in previous research on individuals with dermatitis. In the previous studies by Boonchai et al., the mean DLQI score of 9.5, and by Bhatia et al. and Nagpal et al., the DLQI score ranged from 8.3 to 15.1 [6,15,16]. In contrast, a study by Korkmaz and Boyvat had a higher DLQI score with a mean DLQI score of 13.67 ± 5.88 [11].

Although the present study has provided important insight regarding the CD, it still has a few limitations that need to be discussed so that future studies can be planned more efficiently. The first limitation of the study is the small sample size. Other limitations include a single-centric study, a lack of long-term follow-up, a single tool to assess QoL, a limited number of allergens on the standard test kits, the risk of cross-sensitization, and the effects of confounding variables like systemic diseases and environmental factors that were not studied.

## Conclusions

The present study was an observational and cross-sectional study that analyzed the clinical-epidemiological profile and impairment of QoL in CD. We identified the possible allergens involved in cosmetic CD by patch testing with the Indian cosmetic and fragrance series. The most common cosmetics were hair dye, moisturizer/lotion/cream, and soap/shampoo/cleanser. The most prevalent site of allergy was the face, followed by the scalp, eyelids, and neck. The most common allergen was para-phenylenediamine, followed by the fragrance mix, Kathon CG. We also assessed the impact on the quality of life in patients with cosmetic dermatitis. Many of the patients had mild to moderate DLQI scores. Further multicentric studies with ample sample sizes are required to validate the findings of this study.

## Figures and Tables

**Figure 10:**
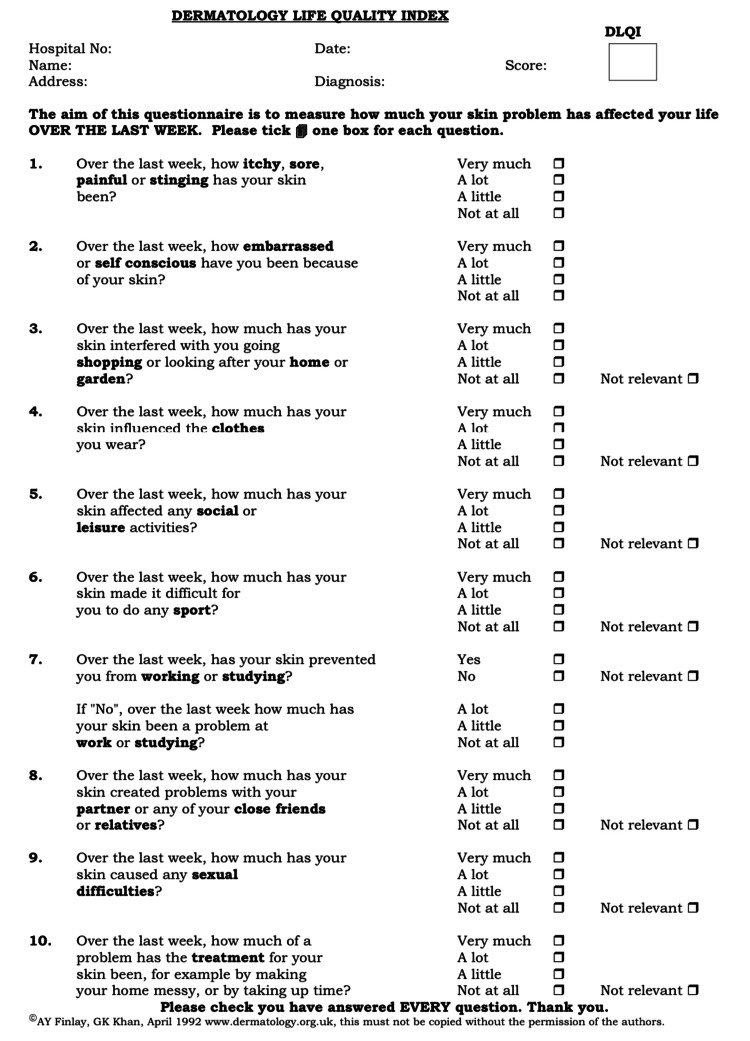
DLQI questionnaire DLQI: Dermatology Life Quality Index

## Appendices

Appendix 1
